# N-Terminal pro-Brain Natriuretic Peptide and Associations With Brain Magnetic Resonance Imaging (MRI) Features in Middle Age: The CARDIA Brain MRI Study

**DOI:** 10.3389/fneur.2018.00307

**Published:** 2018-05-07

**Authors:** Ian T. Ferguson, Martine Elbejjani, Behnam Sabayan, David R. Jacobs, Osorio Meirelles, Otto A. Sanchez, Russell Tracy, Nick Bryan, Lenore J. Launer

**Affiliations:** ^1^Intramural Research Program, National Institute on Aging, National Institutes of Health, Bethesda, MD, United States; ^2^Department of Neurology, Northwestern University, Chicago, IL, United States; ^3^Division of Epidemiology and Community Health, School of Public Health, University of Minnesota, Minneapolis, MN, United States; ^4^Division of Renal Diseases and Hypertension, Department of Internal Medicine, University of Minnesota, Minneapolis, MN, United States; ^5^Department of Pathology and Laboratory Medicine, University of Vermont College of Medicine, Burlington, VT, United States; ^6^Department of Biochemistry, University of Vermont College of Medicine, Burlington, VT, United States; ^7^Department of Radiology, University of Pennsylvania, Philadelphia, PA, United States

**Keywords:** N-terminal pro-brain natriuretic peptide, magnetic resonance imaging, brain volume, cognitive function, middle age, heart–brain axis

## Abstract

**Objective:**

As part of research on the heart–brain axis, we investigated the association of N-terminal pro-brain natriuretic peptide (NT-proBNP) with brain structure and function in a community-based cohort of middle-aged adults from the Brain Magnetic Resonance Imaging sub-study of the Coronary Artery Risk Development in Young Adults (CARDIA) Study.

**Approach and results:**

In a cohort of 634 community-dwelling adults with a mean (range) age of 50.4 (46–52) years, we examined the cross-sectional association of NT-proBNP to total, gray (GM) and white matter (WM) volumes, abnormal WM load and WM integrity, and to cognitive function tests [the Digit Symbol Substitution Test (DSST), the Stroop test, and the Rey Auditory–Verbal Learning Test]. These associations were examined using linear regression models adjusted for demographic and cardiovascular risk factors and cardiac output. Higher NT-proBNP concentration was significantly associated with smaller GM volume (β = −3.44; 95% CI = −5.32, −0.53; *p* = 0.003), even after additionally adjusting for cardiac output (β = −2.93; 95% CI = −5.32, −0.53; *p* = 0.017). Higher NT-proBNP levels were also associated with lower DSST scores. NT-proBNP was not related to WM volume, WM integrity, or abnormal WM load.

**Conclusion:**

In this middle-aged cohort, subclinical levels of NT-proBNP were related to brain function and specifically to GM and not WM measures, extending similar findings in older cohorts. Further research is warranted into biomarkers of cardiac dysfunction as a target for early markers of a brain at risk.

## Introduction

Several studies suggest a role for cardiac health in processes relating to brain atrophy and cognitive impairment at older age, leading to hypotheses concerning the heart–brain axis (1–4). These hypotheses propose pathways through which cardiac dysfunction affects neurocognitive health, including perturbations in factors regulating cerebral perfusion resulting from blood barrier disruption, altered cardiac output, or atherosclerotic changes, that may, over time, lead to brain atrophy and cognitive impairment (5).

One promising biomarker of this axis is amino-terminal pro-brain natriuretic peptide (NT-proBNP), which is released from proBNP along with the physiological active BNP, in response to ventricular wall shear stress. BNP regulates systemic fluid volume through vasodilatation and natriuresis, in part by antagonizing the renin–angiotensin–aldosterone axis (6). NT-proBNP is more stable than BNP in circulation and is often used clinically as a biomarker for left ventricular dysfunction typically in the setting of heart failure. There is a clear association between heart failure and impaired cognition (7); however, NT-proBNP level is also a subclinical measure of cardiovascular dysfunction and a prognostic indicator of cerebrovascular morbidity and mortality (8, 9).

The direct physiological function and clinical relevance of peripheral BNP release on the central nervous system is not fully understood. However, the presence in the brain of widely distributed receptors of the natriuretic peptides, including B-type, suggests additional pathways between the heart and brain, which may contribute to later age cognitive impairment (10). Consistent with this hypothesis, recent studies in older populations show an association between higher NT-proBNP levels and higher risk of dementia and cognitive decline in older adults (11–13). To date, few studies have evaluated the direct relationship of NT-proBNP to brain characteristics and data are even more scarce for middle age men and women (14, 15). Data from younger populations may be particularly helpful to understand the link between NT-proBNP and brain health, given research showing the importance of mid-life exposures to cardiovascular risk factors for later brain changes and cognitive decline (16). Moreover, younger populations are more likely to present with fewer or less advanced cardiovascular co-morbidities and, therefore, increase the likelihood of identifying earlier associations with NT-proBNP and brain outcomes compared to older populations with more cardiovascular damage.

Here, we investigated the association of NT-proBNP to several magnetic resonance imaging (MRI) measures that have been linked to neurocognitive pathology, including total brain volume, gray matter (GM) volume, white matter (WM) volume, abnormal WM load, and WM microstructural integrity (measured using fractional anisotropy) in a well characterized bi-racial, middle-aged community-based sample participating in the Coronary Artery Risk Development in Young Adults (CARDIA) cohort.

We hypothesized that higher NT-proBNP is associated with an MRI profile reflective of relatively higher load of macro and microstructural alterations. In secondary analyses, we further investigated the association of NT-proBNP to cognitive function and to GM lobar tissue volumes.

## Materials and Methods

### Study Population

This study is based on a cross-sectional analysis of data collected in a subsample of the 25th year follow-up exam participants from the Coronary Artery Risk Development in Young Adults (CARDIA) Study. CARDIA is a longitudinal study started in 1985 to investigate cardiovascular risk factors in a bi-racial (white/black) cohort of community-dwelling adults aged 18–30 years at baseline. Recruitment of the cohort (*n* = 5,115) occurred at sites in four US cities (Birmingham, AL, USA; Chicago, IL, USA; Minneapolis, MN, USA; and Oakland, CA, USA). Participants were then followed for 25 years through seven follow-up study visits (response rate at the Y25 exam was 72%).

At the 25th year of follow-up, participants from three of the four CARDIA study centers (Birmingham, AL, USA; Minneapolis, MN, USA; Oakland, CA, USA) were invited to take part in the CARDIA Brain MRI study. This sub-study aimed to identify structural and functional brain changes associated with cardiovascular risk factor trajectories (16). Excluding those with an MRI contraindication, and those with a body habitus too large for the MRI tube bore, 719 participants were recruited within sex–race groups that aimed to mirror the total CARDIA cohort: 35% (*n* = 252) from Oakland, 41.3% (*n* = 297) from Minnesota, and 23.6% (*n* = 170) from Birmingham.

The CARDIA Brain sub-study was carried out in accordance with the recommendations of site-participating local institutional review boards (IRBs) with written informed consent from all subjects. All subjects gave written informed consent in accordance with the Declaration of Helsinki. The protocol was approved by the IRB covering intramural research at the National Institute on Aging.

### Plasma NT-proBNP Measurement

N-terminal pro-brain natriuretic peptide concentration was measured from Year 25 samples using the proBNP II electrochemiluminescence immunoassay on a Cobas e411 chemistry analyzer (Roche Diagnostics Indianapolis, IN, USA). The measuring range is 5–35,000 pg/mL (defined by the lower detection limit and the maximum of the standard curve). The inter-assay coefficient of variation is less than 5%. All NT-proBNP assays were performed in the Laboratory for Biochemistry Research at the University of Vermont.

### MRI Acquisition and Analysis

As previously described (16), brain scans were acquired using 3-T MRI scanners (Oakland: Siemens 3 T Tim Trio/VB 15 platform; Minneapolis: Siemens 3 T Tim Trio/VB 15 platform; and Birmingham: Philips 3 T Achieva/2.6.3.6 platform) located proximally to each participating study center. Post-scan imaging analysis took place at a central processing center (Biomedical Imaging Analysis, Department of Radiology, University of Pennsylvania). Standardized quality-control measures developed for the Functional Bioinformatics Research Network and the Alzheimer’s disease Neuroimaging Initiative (ANI) were implemented on all images. Eight scans did not pass the quality-control checks and were removed from the analysis.

The preprocessing of the T1-weighted scan involved brain extraction (i.e., the removal of the skull, extracerebral tissues, and cerebellum) using a multi-atlas segmentation method (17), correction of image inhomogeneities (18), and segmentation of the brain parenchyma into GM, WM, and cerebrospinal fluid (19). Total brain volume was obtained by summing GM and WM volumes; total intracranial volume was the sum of GM, WM, and cerebral spinal fluid volumes. The brain was segmented into anatomical regions by transferring expert defined region of interest (ROI) labels on a standard template image space to the subject T1 space through nonlinear registration (20). GM and WM were classified into regions of interest according to the Jakob atlas and further into normal and abnormal and tissue (21). Within each ROI, GM and WM volumetric measurements are calculated.

The brain volumes of interest in this study were total brain volume, total GM volume, and total WM volume, as well as lobar GM volumes in the frontal, temporal, and parietal lobes. Regional GM volumes include abnormal and normal tissue; abnormal tissue volume was very low in our cohort. We also investigated the association of NT-proBNP to abnormal WM volume which includes WM tissue damage due to ischemia, demyelination, inflammation, and damaged penumbra tissue surrounding focal infarcts. Because abnormal WM load was low in our sample, we categorized it into high (top 15% of the abnormal WM distribution) versus no-to-low abnormal WM. We evaluated WM integrity, measured using fractional anisotropy from diffusion tensor imaging with higher values indicating better signal diffusion along WM tracts (16, 22).

### Cognitive Function

Three cognitive function tests were performed as part of the core CARDIA Year 25 exam. All cognitive tests were conducted and scored by a certified technician. A script was used for each test and the tests were performed in the same order across subjects to enhance standardization. The Digit Symbol Substitution Test (DSST) assesses cognitive speed, attention, and concentration (23, 24). The Stroop Test assesses executive function and selective attention (25). The Rey Auditory–Verbal Learning Test assesses learning ability and both short-term and delayed memory (26). All scores were converted to *z*-scores prior to analysis.

### Covariate Measures

In our analysis, we accounted for the following potential confounding variables, measured at the Year 25 exam, which were associated with either NT-proBNP and brain volumes in our sample or in prior literature: age, sex, race, total intracranial volume (to correct for head size), education (high defined as >12 years of education, indicating an educational attainment higher than high school, or low ≤12), smoking status (non-smoker, ex-smoker, or current smoker), body mass index (BMI, kg/m^2^), systolic and diastolic blood pressure measurements (average of the second and third blood pressure readings), total cholesterol (mg/dL), glomerular filtration rate (GFR) [estimated using a standard equation that includes serum creatinine concentration, sex, age, and race (27)], diabetes (defined as fasting glucose ≥126 mg/dL or 2 h glucose tolerance test ≥200 mg/dL or hemoglobin A1c ≥6.5% or self-report of taking diabetes medication, all in the absence of pregnancy), hypertension medication use (self-reported at Year 25), renal history (defined as any self-reported history of kidney problems, or diagnosis of nephritis), cardiovascular history (defined as any self-reported heart problems, heart or stroke medication use, or any history of heart problems, heart attack, angina, stroke, or heart failure), and study center. Due to the low frequency of reported cardiac problems in this younger sample, we combined the different cardiovascular disease conditions into one indicator of having history of cardiovascular history versus no history. Missing covariate data (or answers of “unsure”) (*n* = 14) were replaced with values acquired from a previous exam.

### Cardiac Output

In sensitivity analyses, we further adjusted for cardiac output to assess whether the associations between NT-proBNP and brain volumes were independent of a more direct measure of perfusion capacity. Cardiac output measurements were acquired at the Year 25 exam by trained sonographers using a standardized protocol for echocardiogram collection. Measurements were made at the Johns Hopkins University using a standard software analysis system (Digisonics, Houston, TX, USA) (28).

### Analytical Sample

Of the 719 subjects participating in the Year 25 MRI exam, 654 had plasma samples drawn at the same exam the MRI was acquired. Of those, 634 subjects had successfully assayed NT-proBNP levels and successfully processed structural MRI scans and these constituted our analytical sample for the brain volume analyses. Among these individuals, five had clinical levels of >400 pg/mL. Cardiac output was measured in a subset of 564 participants. Fractional anisotropy was measured in subset of 626 participants, and of those, 556 had cardiac output data.

### Statistical Analyses

Descriptive measures are reported for the whole sample and across tertiles of NT-proBNP. Differences in covariates across NT-proBNP tertiles were tested with chi-square for categorical variables and analysis of variance for continuous variables. We used ordinary least squares linear regression models to estimate the associations of NT-proBNP to total brain, total GM, and WM volumes, as well as regional brain volumes, WM integrity, and cognition. Logistic regression models were used for abnormal WM load to compare the top 15% of the sample with the rest of the cohort.

The distribution of NT-proBNP levels was right-skewed (Figure S1 in Supplementary Material) and the distribution of residuals was non-normal when regressing brain volumes on NT-proBNP; therefore, we used natural log transformation for NT-proBNP in all regression models. Brain volumes were normally distributed in our sample.

Three models were examined: Model 1 adjusted for sex, age, race, and total intracranial volume. Model 2 additionally adjusted for potential confounders: education level, smoking status, body mass index, hypertension medication use, systolic blood pressure, diastolic blood pressure, history of diabetes mellitus, history of cardiovascular events, history of renal problems, GFR, total cholesterol, and study center. Model 3 adjusted for all covariates in Model 2 plus cardiac output measured by echocardiography at the Year 25 exam. As Model 3 was based on 564 participants with cardiac output, we re-ran Models 1 and 2 to verify that conclusions of the main analysis were not different in this subsample. We similarly re-ran Models 1 and 2 with the subset of 556 participants who had WM integrity measured by fractional anisotropy.

To illustrate the main findings, we plotted the sex, age, race, and ICV-adjusted gray matter volume (based on Model 1) that corresponded to the mean NT-proBNP for each decile of NT-proBNP. As our sample and others (29) showed that, compared to men, women had higher concentrations of NT-proBNP and given previously reported sex-differences in cardiac disease, we tested for interactions between sex and NT-proBNP for the brain volumes of interest.

All analyses were performed using R Statistical Software (30), version 3.3.1.

## Results

### Sample Characteristics

The mean age in our sample was 50.4 years with a range of 46–52 years: 52% were female, 61.5% were Caucasian, and 38.5% were African-American (Table 1). Distributions of NT-proBNP were different across these demographic factors with older subjects, women, and Caucasians having higher NT-proBNP levels (Table 1). History of cardiovascular disease was present in 12% of the cohort, history of renal disease in 6.5%, and diabetes in 10.4% (Table 1). Mean (±SD) total brain, total WM, and total GM volume were 985 (±107.1), 466.8 (±59.4), and 518.2 mL (±53.7), respectively.

**Table 1:** Characteristics^a^ of the CARDIA brain magnetic resonance imaging sample (2010–2011) according to N-terminal pro-brain natriuretic peptide (NT-proBNP) tertile.

		NT-proBNP Tertile							
	All *n* = 634	Low *n* = 212	Middle *n* = 211	High *n* = 211	*p*-Value^d^
NT-proBNP (pg/mL)					
Range	5–1891	5–29.9	30.1–56.8	56.9–1891	
Mean (SD)	62.9 (103.36)	20.1 (6.5)	42.9 (7.7)	125.7 (160.9)	

**Sociodemographics**					
Age, mean (SD)	50.4 (3.5)	49.6 (3.7)	50.7 (3.2)	50.7 (3.5)	<0.01
Female, *n* (%)	329 (51.9)	61 (28.8)	118 (55.9)	150 (71.1)	<0.01
White, *n* (%)	390 (61.5)	114 (53.8)	138 (65.4)	138 (65.4)	0.02
Low education (≤12 years), *n* (%)	134 (21.1)	51 (24.1)	40 (19.0)	43 (20.4)	0.41
Current smoker, *n* (%)	102 (16.1)	34 (16.0)	32 (15.2)	36 (17.1)	0.99

**Covariates^b^**					
HTN meds, *n* (%)	145 (22.9)	49 (23.1)	48 (22.7)	48 (22.7)	0.99
Diabetes, *n* (%)	66 (10.4)	21 (9.9)	28 (13.3)	17 (8.1)	0.21
BMI, mean (SD)	28.8 (5.8)	29.5 (5.1)	28.2 (6.0)	28.6 (6.0)	0.06
SBP, mean (SD)	118.3 (14.9)	119.7 (12.4)	116.9 (14.7)	118.2 (17.1)	0.15
DBP, mean (SD)	73.6 (10.9)	75.3 (9.8)	73.0 (11.0)	72.6 (11.7)	0.03
Total cholesterol, mean (SD)	192.3 (35.2)	194.0 (33.6)	191.1 (34.2)	191.6 (37.6)	0.66
Cardiovascular disease, *n* (%)	76 (12.0)	24 (11.3)	21 (10.0)	31 (14.7)	0.30
Kidney disease, *n* (%)	41 (6.5)	13 (6.1)	12 (5.7)	16 (7.6)	0.71
GFR (mL/min/1.73^2^), mean (SD)	94.9 (19.5)	96.3 (19.2)	96.5 (17.6)	92.1 (21.3)	0.03
Cardiac output^c^ (L/min), mean (SD)	5.71 (1.58)	5.84 (1.63)	5.36 (1.60)	5.63 (1.49)	0.15

*^a^Unadjusted values of the variables*.

*^b^See Materials and Methods for definitions of covariates*.

*^c^Number of participants with cardiac output measures (*N* = 564, Low = 190, Middle = 192, High = 182)*.

*^d^Reports p-value from ANCOVA for continuous measures or χ^2^ for differences across tertiles*.

*HTN meds, participants on anti-hypertensive medications; BMI, body mass index; SBP, systolic blood pressure; DBP, diastolic blood pressure; GFR, glomerular filtration rate*.

### Primary Results

Higher NT-proBNP concentrations were associated with smaller total GM volume (β = −3.44; 95% CI = −5.68, −1.20), but not with total WM volume (β = 0.45; 95% CI = −2.13, 3.029) after adjusting for potential confounders in both Model 1 and the fully adjusted Model 2 (Table 2). Based on Model 2, each unit higher log (NT-proBNP) concentration was associated with a 3.441-mL smaller total GM volume (Table 2). This means that a 10% higher serum NT-proBNP level is associated with a 0.328 mL (log (1.1)*3.441) smaller GM volume. The association between NT-proBNP and total GM volume was attenuated slightly, but remained statistically significant, after including cardiac output (Table 2, Model 3).

**Table 2:** Log(NT-proBNP) and global brain volumes in the CARDIA brain magnetic resonance imaging sample.

Dependent variable	β^a^	95% CI	*p*-Value
**Total brain volume (mL)**			
Model 1	−2.73	−5.69, 0.23	0.071
Model 2	−2.99	−5.98, −0.0047	0.050
Model 3	−2.31	−5.50, 0.89	0.156

**Total gray matter (mL)**			
Model 1	−3.41	−5.63, −1.19	0.003
Model 2	−3.44	−5.68, −1.20	0.003
Model 3	−2.93	−5.32, −0.53	0.017

**Total white matter (mL)**			
Model 1	0.68	−1.84, 3.19	0.60
Model 2	0.45	−2.13, 3.029	0.73
Model 3	0.62	−2.14, 3.38	0.66

**Abnormal white matter load^b^**			
Model 1	0.02	−0.30, 0.34	0.88
Model 2	−0.12	−0.46, 0.22	0.50
Model 3	−0.02	−0.38, 0.34	0.92

**White matter integrity^c^**			
Model 1	−0.001	−3.48, 0.0009	0.25
Model 2	−0.001	−3.34, 0.001	0.30
Model 3	−0.0007	−0.003, 0.002	0.57

*Model 1: adjusted for age, sex, race, and intracranial volume*.

*Model 2: Model 1 covariates + smoking status, anti-hypertension medication use, systolic blood pressure, diastolic blood pressure, cardiovascular history, renal history, body mass index, total cholesterol, glomerular filtration rate, education, exam center*.

*Model 3: Model 2 covariates + cardiac output*.

*^a^Coefficient represents the difference in brain tissue volume (milliliter) corresponding to 1 unit increase in logNT-proBNP (picograms/milliliter)*.

*^b^Logistic regression models with outcome as dichotomous variable split into high (top 15% of cohort) versus low (bottom 85% of cohort)*.

*^c^Measured by fractional anisotropy*.

**Figure 1:**
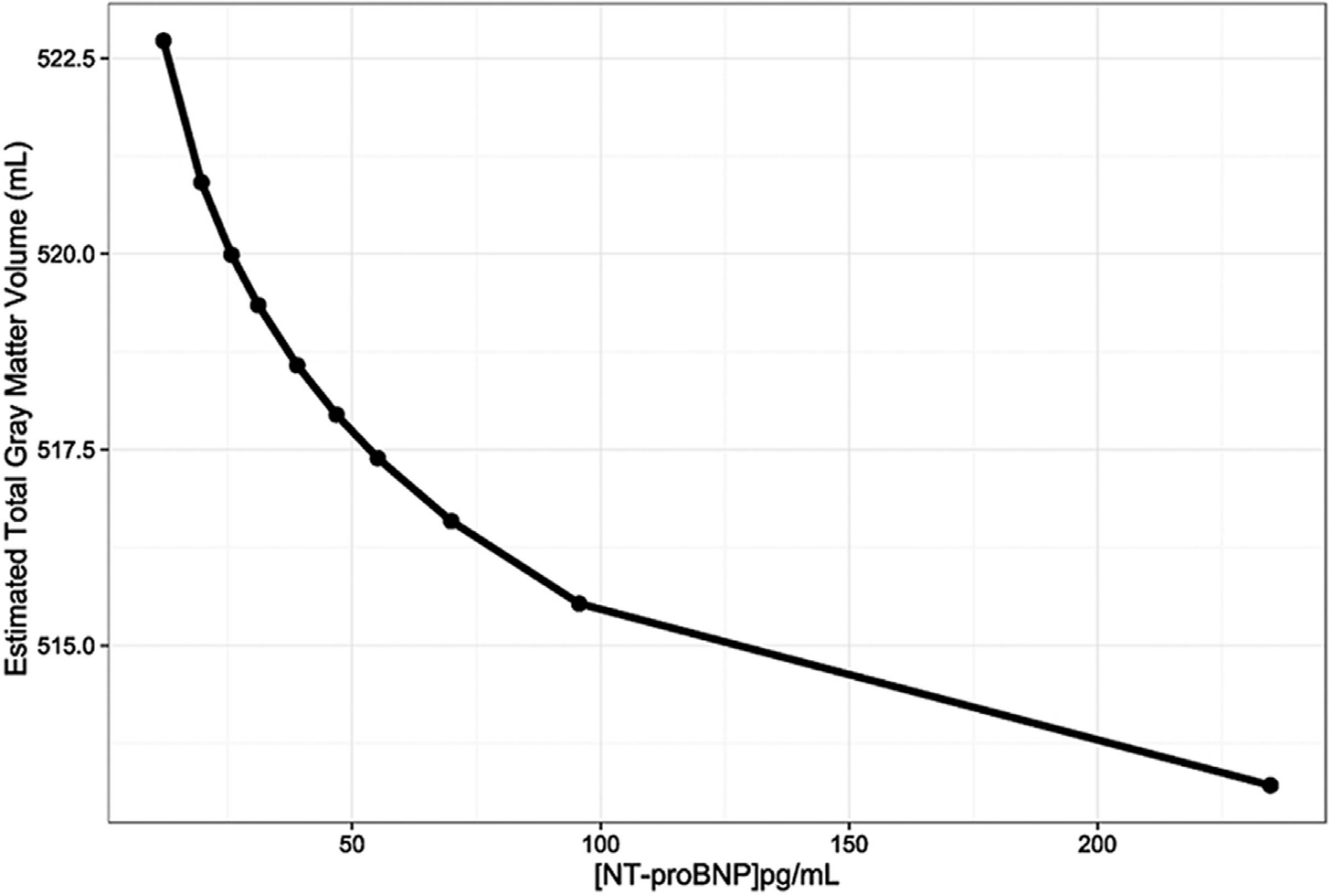
The relationship between gray matter volumes (mL) and N-terminal pro-brain natriuretic peptide (NT-proBNP) levels (pg/mL) adjusted for age, sex, race, and total intracranial volume (Model 1). The line was constructed by connecting the 10 total gray matter volume adjusted estimates for each NT-proBNP concentration that corresponds to the means of NT-proBNP deciles.

This inverse relationship of NT-proBNP with GM volume is illustrated in Figure 1, which represents the adjusted GM volume, based on Model 1, at each of the NT-proBNP values that correspond to the mean of each of the NT-proBNP deciles.

In sensitivity analyses, removal of the five subjects with NT-proBNP concentrations greater than 400 pg/mL (indicating potential heart failure) did not substantially change the association between NT-proBNP and total GM (β = −3.37; *p* = 0.0061 when >400 pg/mL values removed versus β = −3.44; *p* = 0.003 in Model 2). Results of Models 1 and 2 were attenuated but remained significant at *p* < 0.02 when they were rerun on the subsample of 564 with cardiac output data (β = −3.04; *p* = 0.01 in Model 2).

The association of NT-proBNP and total GM volume was not significantly different between men and women (*p* = 0.42 for interaction term in Model 1). We additionally explored whether the association was modified by race, body mass index, and renal function, given their potentially important relationships to NT-proBNP (31–33), and found no indication of effect modification (*p* for interaction terms > 0.27).

There was no association between NT-proBNP and abnormal WM load (OR = 0.88; 95% CI = 0.63, 1.24; Table 2) or WM integrity (β = −0.001; 95% CI = −3.34, 0.001; Table 2). Conclusions were similar in the model adjusted for cardiac output (Table 2, Model 3).

### Secondary Results

Higher NT-proBNP levels were associated with smaller temporal GM volume in the fully adjusted model (β = −1.27; 95% CI = −2.11, −0.43; Table 3, Model 2) and—although slightly attenuated—after adjustment for cardiac output (β = −1.10; 95% CI = −1.20, −0.21; Table 3, Model 3). There were marginal associations between higher NT-proBNP and smaller gray matter volumes in the frontal and parietal lobes (*p* = 0.06 in Model 2 for both), which were attenuated when cardiac output was taken into account (*p* > 0.1 in Model 3).

**Table 3:** Log(NT-proBNP) and lobar gray matter volume in the CARDIA brain magnetic resonance imaging sample.

Dependent variable	β^a^	95% CI	*p*-Value
**Temporal lobe gray matter (mL)**			
Model 1	−1.31	−2.13, −0.49	0.002
Model 2	−1.27	−2.11, −0.43	0.003
Model 3	−1.10	−1.20, −0.21	0.015

**Frontal lobe gray matter (mL)**			
Model 1	−0.94	−1.84, −0.032	0.043
Model 2	−0.90	−1.83, 0.035	0.059
Model 3	−0.75	−1.76, 0.28	0.144

**Parietal lobe gray matter (mL)**			
Model 1	−0.54	−1.10, 0.03	0.063
Model 2	−0.57	−1.15, 0.014	0.056
Model 3	−0.52	−1.14, 0.11	0.104

**Occipital lobe gray matter (mL)**			
Model 1	−0.26	−0.84, 0.31	0.36
Model 2	−0.28	−0.82, 0.25	0.30
Model 3	−0.19	−0.75, 0.38	0.51

*Model 1: adjusted for age, sex, race, and intracranial volume*.

*Model 2: Model 1 covariates + smoking status, anti-hypertension medication use, systolic blood pressure, diastolic blood pressure, cardiovascular history, renal history, body mass index, total cholesterol, glomerular filtration rate, education, and exam center*.

*Model 3: Model 2 covariates + cardiac output*.

*^a^Coefficient represents the difference in brain tissue volume (milliliter) corresponding to 1 unit increase in log NT-proBNP (picograms/milliliter)*.

Association with cognitive function: Higher NT-proBNP levels were associated with significantly lower DSST scores (β = −0.12; 95% CI = −0.21, −0.03; Table 4, Model 2) and showed a trend of association with worst Stroop scores (*p* = 0.05 and 0.01 in Model 1 and 2, respectively).

**Table 4:** Log(NT-proBNP) and cognitive function in the CARDIA brain magnetic resonance imaging sample.

Dependent variable	β^a^	95% CI	*p*-Value
**DSST**			
Model 1	−0.12	−0.22, −0.028	0.01
Model 2	−0.12	−0.21, −0.031	0.01

**STROOP**			
Model 1	0.087	−0.0001, 0.17	0.05
Model 2	0.07	−0.018, 0.16	0.12

**Rey Auditory–Verbal Learning Test**			
Model 1	−0.004	−0.099, 0.09	0.93
Model 2	−0.01	−0.106, 0.08	0.83

*Model 1: adjusted for age, sex, and race*.

*Model 2: Model 1 covariates + smoking status, anti-hypertension medication use, systolic blood pressure, diastolic blood pressure, cardiovascular history, renal history, body mass index, total cholesterol, glomerular filtration rate, education, and exam center*.

*^a^Coefficient indicates the difference in z-scores of the cognitive scores; lower scores on the Digit Symbol Substitution Test (DSST), the Rey Auditory–Verbal Learning Test, and the composite score and higher scores on the STROOP test indicate poorer cognitive functioning*.

## Discussion

In this community-based middle-aged cohort, we found that higher serum NT-proBNP concentration is significantly associated with smaller total GM volume, adjusting for cardiovascular risk factors and cardiac output. Secondary analyses suggested the associations were diffuse across the frontal, parietal, and temporal lobe GM, with relatively stronger results in the temporal lobe. We also found an association between NT-proBNP and lower processing speed scores on the DSST. In contrast, NT-proBNP levels were not associated with WM tissue volume, WM abnormalities, or WM integrity, and the association with NT-proBNP and GM volume was not different in men and women.

There is an accumulating body of literature linking NT-proBNP to various MRI markers for subclinical brain injury including cortical volumes, silent brain infarcts, WM hyperintensities, and cortical cerebral microinfarcts (14, 15, 29, 34–36). More recently, a few studies have investigated the associations of NT-proBNP with earlier and more general brain features and have reported that the strongest associations are with gray matter volume (36, 37). The present study contributes to this literature by reporting a strong association with gray matter, and no association in WM volume or integrity in a cohort with the youngest mean age yet, and that is thought to already present important indicators of the risk of pathologic cascade to dementia. Although no direct conclusions can be drawn about risk of cognitive decline following this analysis, our observation that the strongest GM association was observed with in the temporal lobe is concordant with observations suggesting that the medial temporal lobe gray matter is the locus of greatest atrophy in cognitively intact individuals at high risk for dementia (38).

Finally, we found that NT-proBNP levels were associated with lower DSST scores. This finding supports recent observations of a longitudinal association between NT-proBNP and future cognitive impairment (39, 40). In fact, one study observed a longitudinal association with processing speed measured by DSST but not other domains (41).

The mechanisms that link the cardiovascular system with brain structure and function are not well understood, but are being actively investigated (42, 43). Reduced cardiac output or hypertension is hypothesized to mediate reduced cognitive function *via* a loss of cerebral vascular autoregulation resulting in hypoxia and regional tissue atrophy (43, 44). The findings in this study suggest that NT-proBNP concentrations within normative ranges and below diagnostic thresholds for clinically overt heart failure (<400 pg/mL) may be an early marker of dysfunction in both the heart and the brain in middle-aged adults (45, 46). The observation that gray matter may be the first sign of cognitive decline in the heart–brain axis is plausible given evidence suggesting the medial tempo-parietal cortex is preferentially affected by reduced blood flow because of its relatively high metabolic demand (47). Further, although results were attenuated after adjusting for cardiac output, the findings suggest a potentially more direct relationship between NT-proBNP and brain structure beyond its role as marker of myocardial wall stress, possibly related to the presence of BNP receptors on neuronal tissue which may affect cerebral function through the regulation of blood barrier integrity, synaptic transmission, and the modulation of both the systemic and central nervous system stress response (48).

An important consideration for the hypothesis that BNP acts *via* the heart–brain axis to mediate neuronal tissue loss by first being released as a result of cardiac dysfunction is whether NT-proBNP measured in the serum is entirely cardiac derived or is partially derived from neuronally expressed pre-proBNP. Although the majority of BNP-producing tissue is cardiac in origin (49), we are unaware of any investigation which quantifies the relative amount measured in serum. In fact, while BNP is variably found throughout the brain, the lack of mRNA expression in neuronal tissue for the B-type peptide suggests that pre-proBNP may not be synthesized by neuronal tissue at all, and thus all NT-proBNP measured in serum would be derived from cardiac pre-proBNP (50). Characterizing the expression profile of BNP within the CNS, as well as elucidating the specific actions of BNP from the periphery on the CNS, will be important areas of investigation going forward.

### Strengths/Limitations

This study has several strengths. We investigated the association of NT-proBNP and brain structure and function, in a well-described community-based cohort of middle-aged adults. This potentially limits confounding by accumulated exposure to cardiovascular risk factors and cardiovascular disease burden typically observed in older populations. It also reduces the concern for “reverse causality” whereby brain changes lead to changes in cardiovascular risk factors and not the other way around, as is possible in studies of older persons. Other strengths include the community-based sample, which increases the range of health in the sample, and adjustment of several potential confounders, including echocardiography measures of heart function.

Because this study is cross-sectional, we cannot make robust inference on the directionality of the observed association between NT-proBNP and smaller brain volumes. Further, we cannot exclude the possibility that unmeasured confounders (such as genetic or other developmental processes that simultaneously affect both heart and brain health) contributed to the observed association between NT-proBNP and GM volume, even though we adjusted for a number of cardiovascular risk factors. Another issue concerns the fully automated procedures of brain volume estimation, which are optimal for large community-based studies, but can potentially include measurements error due to residual inter-subject variability. However, the errors are unlikely to be systematically related to NT-proBNP and to be driving our results.

### Conclusion

We observed a significant association between higher NT-proBNP concentration and lower total GM volume, but not total WM volume or WM integrity and abnormalities, in a bi-racial cohort of 634 community-dwelling participants with a mean age of 50 years. This association was significant after adjusting for cardiovascular risk factors as well as cardiac output. Higher NT-proBNP concentrations were also associated with poorer processing speed. Our findings highlight a potentially differential relationship between subclinical markers of cardiac health and GM tissue at younger age and motivate further research into biomarkers of cardiac dysfunction as early markers of a brain at risk and into the temporal succession of the deterioration of brain health in relation to cardiac pathology.

## Ethics Statement

The CARDIA brain sub-study was carried out in accordance with the recommendations of site-participating local IRBs with written informed consent from all subjects. All subjects gave written informed consent in accordance with the Declaration of Helsinki. The protocol was approved by the IRB covering intramural research at the National Institute on Aging.

## Author Contributions

IF: analysis and interpretation of data and drafting and revising the manuscript. ME: analysis and interpretation of data and revising the manuscript. BS, RT, and NB: interpretation of data and critical review of the manuscript. DJ and OM: analysis, critical review of the manuscript, and interpretation of data. OS: critical review of the manuscript. LL: conception and study design, analysis and interpretation of data, and revising the manuscript.

## Conflict of Interest Statement

The authors declare that the research was conducted in the absence of any commercial or financial relationships that could be construed as a potential conflict of interest. The reviewers KR and handling Editor declared their shared affiliation.

## Supplementary Material

Figure S1 Distribution of NT-proBNP in CARDIA Brain MRI sample.

## References

[1] Verghese J Lipton RB Hall CB Kuslansky G Katz MJ. Low blood pressure and the risk of dementia in very old individuals. Neurology (2003) 61(12):1667–72. 10.1212/01.WNL.0000098934.18300.BE 14694027

[2] Alves TCDF Busatto GF. Regional cerebral blood flow reductions, heart failure and Alzheimer’s disease. Neurol Res (2006) 28(6):579–87. 10.1179/016164106X130416 16945208

[3] de la Torre JC Cardiovascular risk factors promote brain hypoperfusion leading to cognitive decline and dementia. Cardiovasc Psychiatry Neurol (2012) 2012:367516 10.1155/2012/367516 23243502 PMC3518077

[4] Ritz K van Buchem MA Daemen MJ. The heart-brain connection: mechanistic insights and models. Neth Heart J (2013) 21(2):55–7. 10.1007/s12471-012-0348-9 23179612 PMC3547426

[5] Jefferson AL. Cardiac output as a potential risk factor for abnormal brain aging. J Alzheimers Dis (2010) 20(3):813–21. 10.3233/JAD-2010-100081 20413856 PMC3041147

[6] Dadu RT Nambi V Ballantyne CM. Developing and assessing cardiovascular biomarkers. Transl Res (2012) 159(4):265–76. 10.1016/j.trsl.2012.01.003 22424430

[7] Leto L Feola M. Cognitive impairment in heart failure patients. J Geriatr Cardiol (2014) 11(4):316–28. 10.11909/j.issn.1671-5411.2014.04.007 25593581 PMC4294149

[8] Kistorp C Raymond I Pedersen F Gustafsson F Faber J Hildebrandt P. N-terminal pro-brain natriuretic peptide, C-reactive protein, and urinary albumin levels as predictors of mortality and cardiovascular events in older adults. JAMA (2005) 293(13):1609–16. 10.1001/jama.293.13.1609 15811980

[9] Pedersen F Raymond I Kistorp C Sandgaard N Jacobsen P Hildebrandt P. N-terminal pro-brain natriuretic peptide in arterial hypertension: a valuable prognostic marker of cardiovascular events. J Card Fail (2005) 11(5 Suppl):S70–5. 10.1016/j.cardfail.2005.04.015 15948105

[10] Pemberton CJ Yandle TG Espiner EA. Immunoreactive forms of natriuretic peptides in ovine brain: response to heart failure. Peptides (2002) 23(12):2235–44. 10.1016/S0196-9781(02)00263-2 12535704

[11] Daniels LB Laughlin GA Kritz-Silverstein D Clopton P Chen WC Maisel AS Elevated natriuretic peptide levels and cognitive function in community-dwelling older adults. Am J Med (2011) 124(7):670.e671–678. 10.1016/j.amjmed.2011.02.027 21683832 PMC3173742

[12] Wijsman LW Sabayan B van Vliet P Trompet S de Ruijter W Poortvliet RK N-terminal pro-brain natriuretic peptide and cognitive decline in older adults at high cardiovascular risk. Ann Neurol (2014) 76(2):213–22. 10.1002/ana.24203 24942833

[13] Sabayan B van Buchem MA Sigurdsson S Zhang Q Harris TB Gudnason V Cardiac hemodynamics are linked with structural and functional features of brain aging: the age, gene/environment susceptibility (AGES)-Reykjavik Study. J Am Heart Assoc (2015) 4(1):e001294. 10.1161/JAHA.114.001294 25628405 PMC4330056

[14] Dadu RT Fornage M Virani SS Nambi V Hoogeveen RC Boerwinkle E Cardiovascular biomarkers and subclinical brain disease in the atherosclerosis risk in communities study. Stroke (2013) 44(7):1803–8. 10.1161/STROKEAHA.113.001128 23660848 PMC4334904

[15] Pikula A Beiser AS DeCarli C Himali JJ Debette S Au R Multiple biomarkers and risk of clinical and subclinical vascular brain injury: the Framingham Offspring Study. Circulation (2012) 125(17):2100–7. 10.1161/CIRCULATIONAHA.110.989145 22456473 PMC3427730

[16] Launer LJ Lewis CE Schreiner PJ Sidney S Battapady H Jacobs DR Vascular factors and multiple measures of early brain health: CARDIA Brain MRI Study. PLoS One (2015) 10(3):e0122138. 10.1371/journal.pone.0122138 25812012 PMC4374951

[17] Doshi J Erus G Ou Y Gaonkar B Davatzikos C. Multi-atlas skull-stripping. Acad Radiol (2013) 20(12):1566–76. 10.1016/j.acra.2013.09.010 24200484 PMC3880117

[18] Sled JG Zijdenbos AP Evans AC. A nonparametric method for automatic correction of intensity nonuniformity in MRI data. IEEE Trans Med Imaging (1998) 17(1):87–97. 10.1109/42.668698 9617910

[19] Li C Gore JC Davatzikos C. Multiplicative intrinsic component optimization (MICO) for MRI bias field estimation and tissue segmentation. Magn Reson Imaging (2014) 32(7):913–23. 10.1016/j.mri.2014.03.010 24928302 PMC4401088

[20] Shen D Davatzikos C. HAMMER: hierarchical attribute matching mechanism for elastic registration. IEEE Trans Med Imaging (2002) 21(11):1421–39. 10.1109/TMI.2002.803111 12575879

[21] Collins DL Zijdenbos AP Kollokian V Sled JG Kabani NJ Holmes CJ Design and construction of a realistic digital brain phantom. IEEE Trans Med Imaging (1998) 17(3):463–8. 10.1109/42.712135 9735909

[22] Le Bihan D Mangin JF Poupon C Clark CA Pappata S Molko N Diffusion tensor imaging: concepts and applications. J Magn Reson Imaging (2001) 13(4):534–46. 10.1002/jmri.1076 11276097

[23] Beck AT Feshback S Legg D The clinical utility of the digit symbol test. J Consult Psychol (1962) 26:263–8. 10.1037/h0049298 13866248

[24] Wechsler D The Measurement of Adult Intelligence. Baltimore: Williams & Wilkins (1939).

[25] Stroop J Studies of interference in serial verbal reactions. J Exp Psychol (1935) 18:643–62. 10.1037/h0054651

[26] Rosenberg SJ Ryan JJ Prifitera A. Rey auditory-verbal learning test performance of patients with and without memory impairment. J Clin Psychol (1984) 40(3):785–7. 10.1002/1097-4679(198405)40:3<785::AID-JCLP2270400325>3.0.CO;2-4 6746989

[27] Levey AS Stevens LA Schmid CH Zhang YL Castro AF III Feldman HI A new equation to estimate glomerular filtration rate. Ann Intern Med (2009) 150(9):604–12. 10.7326/0003-4819-150-9-200905050-00006 19414839 PMC2763564

[28] Kishi S Teixido-Tura G Ning H Venkatesh BA Wu C Almeida A Cumulative blood pressure in early adulthood and cardiac dysfunction in middle age: the CARDIA study. J Am Coll Cardiol (2015) 65(25):2679–87. 10.1016/j.jacc.2015.04.042 26112189

[29] Sabayan B van Buchem MA de Craen AJ Sigurdsson S Zhang Q Harris TB N-terminal pro-brain natriuretic peptide and abnormal brain aging: The AGES-Reykjavik Study. Neurology (2015) 85(9):813–20. 10.1212/WNL.0000000000001885 26231259 PMC4553023

[30] R Development Core Team R: A Language and Environment for Statistical Computing [Computer Program]. Vienna, Austria (2015).

[31] Gupta DK de Lemos JA Ayers CR Berry JD Wang TJ. Racial differences in natriuretic peptide levels: the Dallas Heart Study. JACC Heart Fail (2015) 3(7):513–9. 10.1016/j.jchf.2015.02.008 26071618 PMC4498971

[32] Zheng LH Wu LM Yao Y Chen WS Bao JR Huang W Impact of body mass index on plasma N-terminal ProB-type natriuretic peptides in Chinese atrial fibrillation patients without heart failure. PLoS One (2014) 9(8):e105249. 10.1371/journal.pone.0105249 25144363 PMC4140742

[33] Anwaruddin S Lloyd-Jones DM Baggish A Chen A Krauser D Tung R Renal function, congestive heart failure, and amino-terminal pro-brain natriuretic peptide measurement: results from the ProBNP Investigation of Dyspnea in the Emergency Department (PRIDE) Study. J Am Coll Cardiol (2006) 47(1):91–7. 10.1016/j.jacc.2005.08.051 16386670

[34] Vilar-Bergua A Riba-Llena I Penalba A Cruz LM Jiménez-Balado J Montaner J N-terminal pro-brain natriuretic peptide and subclinical brain small vessel disease. Neurology (2016) 87(24):2533–9. 10.1212/WNL.0000000000003423 27956564

[35] Hilal S Chai YL van Veluw S Shaik MA Ikram MK Venketasubramanian N Association between subclinical cardiac biomarkers and clinically manifest cardiac diseases with cortical cerebral microinfarcts. JAMA Neurol (2017) 74(4):403–10. 10.1001/jamaneurol.2016.5335 28166312 PMC5470359

[36] Zonneveld HI Ikram MA Hofman A Niessen WJ van der Lugt A Krestin GP N-terminal pro-B-type natriuretic peptide and subclinical brain damage in the general population. Radiology (2017) 283(1):205–14. 10.1148/radiol.2016160548 27924720

[37] Sabayan B van Buchem MA Sigurdsson S Zhang Q Meirelles O Harris TB Cardiac and carotid markers link with accelerated brain atrophy: the AGES-Reykjavik Study (age, gene/environment susceptibility-reykjavik). Arterioscler Thromb Vasc Biol (2016) 36(11):2246–51. 10.1161/ATVBAHA.116.308018 27609370 PMC5310810

[38] Knopman DS Jack CR Jr Lundt ES Wiste HJ Weigand SD Vemuri P Role of beta-amyloidosis and neurodegeneration in subsequent imaging changes in mild cognitive impairment. JAMA Neurol (2015) 72(12):1475–83. 10.1001/jamaneurol.2015.2323 26437123 PMC4735877

[39] Cushman M Callas PW McClure LA Unverzagt FW Howard VJ Gillett SR N-terminal pro-B-type natriuretic peptide and risk of future cognitive impairment in the REGARDS cohort. J Alzheimers Dis (2016) 54(2):497–503. 10.3233/JAD-160328 27567834

[40] Tynkkynen J Hernesniemi JA Laatikainen T Havulinna AS Salo P Blankenberg S High-sensitivity cardiac troponin I and NT-proBNP as predictors of incident dementia and Alzheimer’s disease: the FINRISK Study. J Neurol (2017) 264(3):503–11. 10.1007/s00415-016-8378-7 28039523

[41] Mirza SS de Bruijn RF Koudstaal PJ van den Meiracker AH Franco OH Hofman A The N-terminal pro B-type natriuretic peptide, and risk of dementia and cognitive decline: a 10-year follow-up study in the general population. J Neurol Neurosurg Psychiatry (2016) 87(4):356–62. 10.1136/jnnp-2014-309968 25918047

[42] Dardiotis E Giamouzis G Mastrogiannis D Vogiatzi C Skoularigis J Triposkiadis F Cognitive impairment in heart failure. Cardiol Res Prac (2012) 2012:595821. 10.1155/2012/595821 22720185 PMC3375144

[43] Jefferson AL Beiser AS Himali JJ Seshadri S O’Donnell CJ Manning WJ Low cardiac index is associated with incident dementia and Alzheimer disease: the Framingham Heart Study. Circulation (2015) 131(15):1333–9. 10.1161/CIRCULATIONAHA.114.012438 25700178 PMC4398627

[44] Qiu C von Strauss E Fastbom J Winblad B Fratiglioni L. Low blood pressure and risk of dementia in the Kungsholmen project: a 6-year follow-up study. Arch Neurol (2003) 60(2):223–8. 10.1001/archneur.60.2.223 12580707

[45] Mitchell A Misialek JR Folsom AR Duprez D Alonso A Jerosch-Herold M Usefulness of N-terminal pro-brain natriuretic peptide and myocardial perfusion in asymptomatic adults (from the multi-ethnic study of atherosclerosis). Am J Cardiol (2015) 115(10):1341–5. 10.1016/j.amjcard.2015.02.040 25816778 PMC4414796

[46] Kerola T Nieminen T Hartikainen S Sulkava R Vuolteenaho O Kettunen R B-type natriuretic peptide as a predictor of declining cognitive function and dementia – a cohort study of an elderly general population with a 5-year follow-up. Ann Med (2010) 42(3):207–15. 10.3109/07853891003652542 20384435

[47] Ruitenberg A den Heijer T Bakker SL van Swieten JC Koudstaal PJ Hofman A Cerebral hypoperfusion and clinical onset of dementia: the Rotterdam Study. Ann Neurol (2005) 57(6):789–94. 10.1002/ana.20493 15929050

[48] Cao LH Yang XL. Natriuretic peptides and their receptors in the central nervous system. Prog Neurobiol (2008) 84(3):234–48. 10.1016/j.pneurobio.2007.12.003 18215455

[49] Gutkowska J Antunes-Rodrigues J McCann SM. Atrial natriuretic peptide in brain and pituitary gland. Physiol Rev (1997) 77(2):465–515. 10.1152/physrev.1997.77.2.465 9114821

[50] Hodes A Lichtstein D. Natriuretic hormones in brain function. Front Endocrinol (2014) 5:201. 10.3389/fendo.2014.00201 25506340 PMC4246887

