# Supplementary figures and images for: N-Terminal pro-Brain Natriuretic Peptide and Associations With Brain Magnetic Resonance Imaging (MRI) Features in Middle Age: The CARDIA Brain MRI Study
Source: Front Neurol. 2018 May 7;9:307. doi: 10.3389/fneur.2018.00307 (PMC5949318; doi:10.3389/fneur.2018.00307)

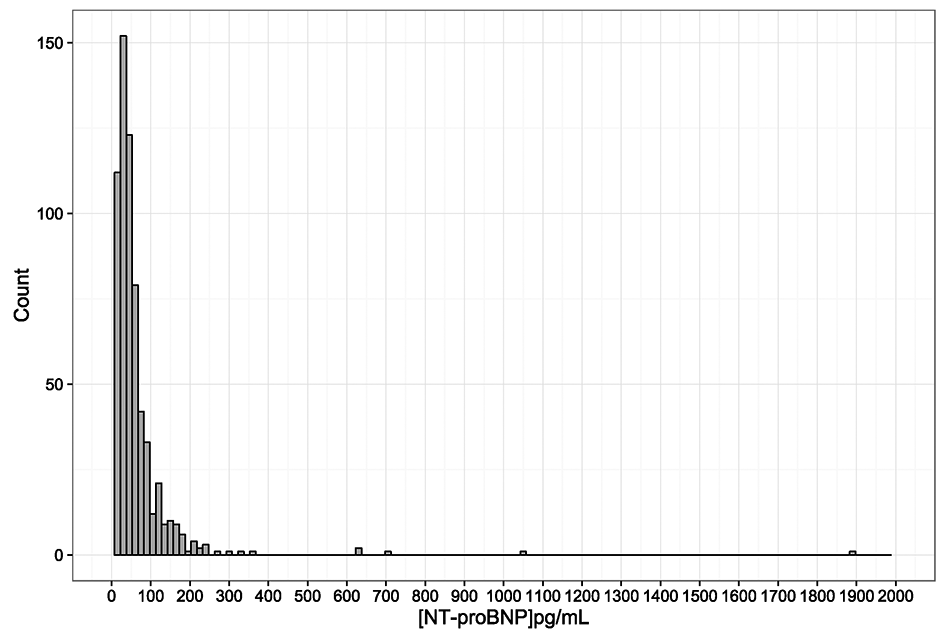

Supplement: Figure S1 — Distribution of NT-proBNP in CARDIA Brain MRI sample. [file Image_1.tiff]
